# Synthesis, characterisation and cytotoxicity of gold microwires for ultra-sensitive biosensor development

**DOI:** 10.1186/s12934-020-01478-y

**Published:** 2021-02-17

**Authors:** Nurul Akmal Che Lah, Robert Gray, Sonia Trigueros

**Affiliations:** 1 Innovative Manufacturing, Mechatronics and Sports Lab (iMAMS), Faculty of Manufacturing and Mechatronics Engineering Technology, University Malaysia Pahang, 26600 Pekan, Pahang Malaysia; 2 University College London, Gower St, Bloomsbury, London, WC1E 6BT UK; 3 Department of Zoology, University of Oxford, Oxford, OX1 3PS UK

**Keywords:** Biosensor, Biomarkers, Antimicrobial, Cytotoxicity, Gold microwires

## Abstract

With the long-term goal of developing an ultra-sensitive microcantilever-based biosensor for versatile biomarker detection, new controlled bioreceptor-analytes systems are being explored to overcome the disadvantages of conventional ones. Gold (Au) microwires have been used as a probe to overcome the tolerance problem that occurs in response to changes in environmental conditions. However, the cytotoxicity of Au microwires is still unclear. Here, we examined the cytotoxicity of Au microwires systems using both commercial and as-synthesised Au microwires. In vitro experiments show that commercial Au microwires with an average quoted length of 5.6 µm are highly toxic against Gram-negative *Escherichia coli* (*E. coli*) at 50 µg/mL. However, this toxicity is due to the presence of CTAB surfactant not by the microwires. Conversely, the as-synthesised Au microwires show non-cytotoxicity even at the maximum viable concentration (330 µg/mL). These findings may lead to the development of potentially life-saving cytotoxicity-free biosensors for an early diagnostic of potential diseases.

## Introduction

A wide array of biomarkers secreted by cancerous cells can be found in blood samples [1–6]. With current emerging proteomics technologies, it is straightforward to obtain reliable sets of disease-specific protein biomarkers. Early identification of disease blood protein signatures, is becoming the most promising strategy for effective cancer prevention [7–10]. Nevertheless, some protein biomarkers, such as those secreted by cell death in tumours microenvironment at a very early growth period, are produced only at ultra-low concentrations [11–14]. Low reproducibility highlights the difficulty in detecting low concentrations of disease protein biomarkers. With concentration, the scales are six to seven orders of magnitude lower than the plasma protein (e.g. the reference range of albumin protein in blood is approximately 35–55 µg/L [15, 16]), making their detection challenging. Engineered materials-based biosensors with high sensitivity levels have been the focus of improved biomarker detection technology [17–20]. These advanced detection methods depend on the antibody and protein for recognition, identification and quantification of targeted cells.

There are several drawbacks on microcantilever (MC) biosensor, MC requires a high amount of analytes and should be sensitive enough to measure deflection in the range of few micrometres leads [21, 22] to the integration of microfluid biosensor layer for protein–protein interaction detection [23]. Hence, micromaterial-based MC biosensors have good potential in advanced biosensor technology as they offer a excellent surface-to-volume ratio, so they are able to mediate faster with a higher kinetic electron transfer, allowing the merit of multiple sensing mechanisms in a single platform [18, 24–26]. The size of micromaterials can facilitate effective interaction depending on the nature of the chemical bonding with targeted biomolecules in opaque liquids such as blood or urine [27, 28]. Offering potential for electronic detection surface stress functionalisation as a result of the MC bending by a certain extent generated by analyte–ligand interactions in the biomarker layer.

Gold (Au) based one-dimensional (1-D) micromaterial is a candidate for achieving an ultrasensitive MC biosensor device [28–33]. Specifically, for flexible chemo resistive sensors. Au microwires are attractive because they possess minimal cross-sectional area but have the ability to increase the flood-current along the axial current direction, resulting in higher conductance changes compared to the typical zero-dimensional (0-D) micromaterials [34]. The chemoreceptive characteristics of AuNWs such as the capability to serve as biocompatible surface for the immobilisation of biomolecules in a correct orientation which enhances the binding affinity of antigen–antibody and the ability to support a swift electron-transfer which causes the flow of one-way electron between two electrodes are key considerations in designing the MC microbiosensor [19, 20]. Chemoreceptive and also conductometric characteristics played by the Au microwires are both important for biosensor transduction mechanism.

On the other hand, there have been many concerns about the potential cytotoxicity of micromaterials in general, which may arise upon medical application [35–37]. It has been proved that several micromaterials consisting of heavy metals may release ions that may cause cytotoxic effects. Although Au microwires are not considered toxic in most cases, there is a certain ambiguity about Au microwires toxicity impact regarding variability and threshold for specific cell types [38]. Therefore, there is an urgent need to present new data that can assist in developing a mass-sensitive MC-based Au microwires biosensor. Important primary parameters that are involved in cytotoxicity assessments of Au microwires include the aspect ratio of microwires, surface functionalisation method, cell type, administration of dosage and application protocols. In this work, we describe the synthesis of bio-friendly Au microwires for MC biosensor application (see Additional file 1: Figure S1). The objective is to ascertain the maximum dose of as-synthesised Au microwires which is safe to avoid initial cytotoxicity whilst increasing the sensitivity of the microwires array in comparison with commercial Au microwires suspended in CTAB. Bacteria cells are the most sensitive cells to detect toxicity arising from either free or microstructured metallic ions. For toxicity detection, we use a set of antimicrobial approaches. The transduction mechanism of microsensors depends on the binding of the protein of interest change which yields different conductivity output. The Au microwires play a vital role in the transduction mechanism. Hence it is essential to maximise the concentration of Au microwires to achieve maximum sensitivity. The investigation of the transduction mechanism is not within the scope of this study. The assembling microwires on the top surface of the microscopy probe is also evaluated but not discuss further in the present study and only included in Additional file 1. Any biomarker should be able to be detected using this platform.

## Materials and methods

### Au microwires preparation

Oleylamine, OA (technical grade 70%), triisopropylsilane, TIPS (98%), hexane (CH_3_(CH_2_)_4_CH_3_, anhydrous, 95%) and ethanol (CH_3_CH_2_OH, absolute, ≥ 99.8%) were purchased from Merck Sigma-Aldrich, United Kingdom. All the chemicals were used without further purification.

Commercial AuNWs (dispersion in H_2_O, contains cetyl trimethyl ammonium bromide, CTAB as a stabiliser) used was purchased from Merck Sigma-Aldrich, United Kingdom. The AuNWs is specified to have an average diameter size and length of 30 nm and 4.5 µm, respectively, with a concentration of $\ge$ 50 µg/mL. Nonetheless, the size and length observed under the FESEM are in micrometer size range.

### Microbial cell culture preparation

The commercial *Escherichia coli* (*E. coli*) *DH5-Alpha strain* (*DH5α*), an engineered non-pathogenic strain for routine subcloning procedures is used in the present work. It is quick and easy to grow and has been used extensively as a lab microbial model system. (ThermoFisher catalogue number 18263618). *E. coli* cells were grown in Luria–Bertani medium (LB), rich medium for cell growth. The cells were initially grown from an original culture sample, kept at − 80 °C. Cells were inoculated onto an LB agar plate and incubated at 37 °C for 24 h. At this point, a number of individual colonies had grown, each from a single cell. To reduce variation across the bacteria under study, a single colony was selected and incubated in LB to produce a working cell solution. This was used as the master cell culture for all the bacteria cell experiments. Optical cell density measurements were acquired using a Perkin Elmer Lambda Bio^+^ Spectrophotometer (Germany). The spectrophotometric method measured the optical density at 600 nm based on an American Public Health Association (APHA) standard. The optical density (OD) of 0 is set for pure LB, and the spectrophotometer is calibrated so that the OD equal to 1.00 corresponds to a cell density of 109 ± 5 × 108/mL. A linear relationship between OD and cell density is achieved over this range.

### Cytotoxicity validation methods

#### Disk diffusion assay

Cytotoxicity test is carried out through the agar disk diffusion assay method which has been used in many clinical microbiology laboratories for routine antimicrobial susceptibility testing. The standard measurement is based on the Clinical Laboratory Standard Institute (CLSI) and manufacturers’ standard for each sample. The Au microwires colloidal solution (300 µg/mL) was suspended in distilled water and this portion deposited on the sterile paper disk that is 6 mm in diameter with 4 replicate disk per sample. The disk was placed on Gram-negative *E. coli DH5α* strain. Controls were prepared using pure hexane and/or distilled water.

*Escherichia coli DH5α* were inoculated at a concentration of 107 to 108 cells/mL at stationary phase strain onto Luria–Bertani (LB) agar. The LB agar plates were then inoculated and incubated for 24 h at 37 °C to record the cytotoxicity effect. The size of the inhibition zone around the disc is control by the interchange between the Au microwires diffusion rate (Eq. 1), the exponential growth rate of the bacteria and the minimum inhibitory concentration which is usually higher than the breakpoint.

1 $$ C\left( {r,t} \right) = \frac{{A \cdot e^{{\left( {\frac{{ - r^{2} }}{4Dt}} \right)}} }}{4\pi hDt}. $$

The *C* is referring to the Au microwires concentration at time (*t*) and distance (*r*) from the point source (disc centre) with the diffusively isotropic assumption in a 2-D plate form. The *D* is the corresponding diffusion coefficient and *A* is the amount of initial Au microwires used in the disc with *h* is the height of the agar in the plate. The concentration of Au microwires in the inhibition zone could drop below the minimum inhibitory concentration after overnight incubation. By this time, the diffusion of the nutrient in the plate occurs and gradually depleted by the bacteria that grew in the inhibition zone periphery. Overnight incubation made the cells cover almost 70% of the growing surface, except for the circles of growth surrounding disc which remained clear. The diameters of these circles are then measured and used as measurements of the inhibitory effects of the sample.

#### Colony forming unit counting method (CFU)

Methods for in vitro evaluation of antimicrobial activity, seek to assess the total number of *E. coli* cells in culture media [39]. Colony-forming units (CFU) of *E. coli DH5α* were counted after plating triplicate serial dilutions of LB cultures on LB-Agar plates and incubating overnight at 37 °C. Plates containing between 2 and 200 individually identifiable colonies were counted. The relative number of colonies on a plate compared to the control gives a value which is a measurement of the toxicity.

### Microstructures characterisation methods

#### Characterisation of as-synthesised and commercial Au microwires

Au microwires were resuspended and sonicated in ethanol medium. And let it dry on an aluminium substrate at room temperature. Field emission scanning electron microscopy (FESEM) imaging was conducted by a JEOL JSM 7500 F microscope equipped with a cold field emission gun at an acceleration voltage of 15 kV. An in-lens detector collected secondary electrons and backscattered electrons signals and in-chamber multi-quadrant annular retractable solid-state detector, respectively. Element analysis was carried out using Zeiss Neon 40EsB (Carl Zeiss NTS GmbH, Oberkochen, Germany) at 20 kV equipped with energy dispersive x-ray spectroscopy, EDX (Inca software, Oxford Instruments). Image analysis on FESEM data was carried out using ImageJ 1.50i. Au microwires density was determined using the plugin ‘analyse particles’ function of ImageJ on a total of 20 SEM images of Au microwires samples. The aggregated size of Au microwires was calculated from a total of 2000 randomly picked aggregated Au microwires from FESEM images.

## Results

### Synthesis of Au microwires

**Fig. 1:**
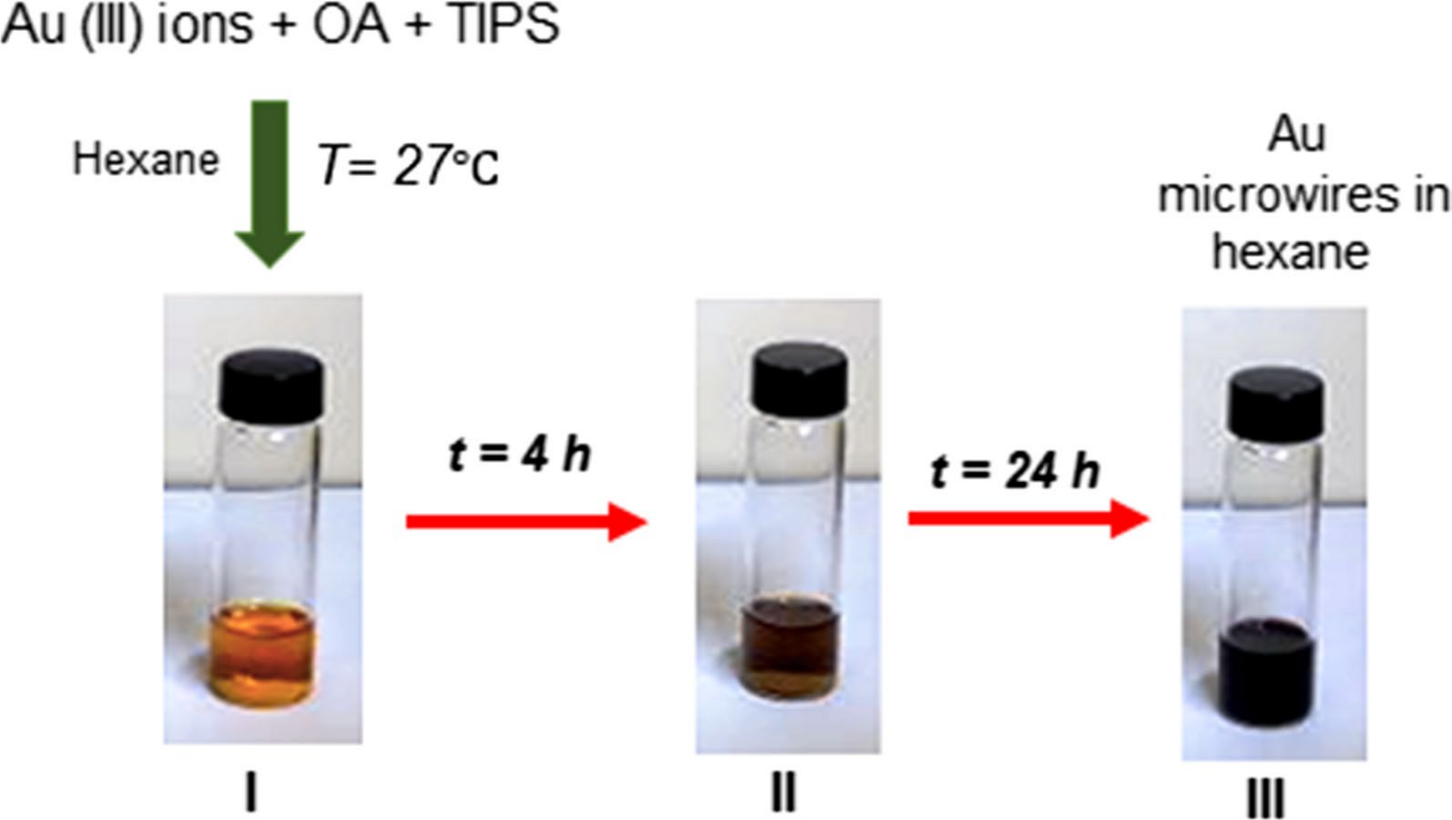
As-synthesised Au microwires reaction formation flow. **I** The solution of HAuCl_4_·3H_2_O + OA + TIPS after 1 min of the mixture. **II** After the mixture is left at room temperature for 4 h and **III** 24 h. The reaction product has a dark violet colour solution after 24 h

Au microwires were synthesised based on the method described in the literature [34]. In the absence of heat, the synthesis reaction involved mixing of 100 µL OA and 150 µL TIPS with about 3 to 5 mg of gold (III) chloride precursor solution, (HAuCl_4_ 99.9% trace metals basis, 30 wt% in dilute HCl) in 2.5 mL final volume of hexane without stirring. OA acts as a stabiliser and a template for 1-D growth with TIPS role as a highly reactive reducing agent. The observation of the reaction through the change in colour from light orange to red, followed by dark violet during the period of reaction left out at room temperature is demonstrated in Fig. 1. The Au microwires products were centrifuged, washed with ethanol at least 3 times and finally re-dispersed in hexane for further characterisations. In this work, the resulting Au microwires were more than 100 nm in diameter with a standard deviation of 0.8%. During the synthesis reaction, the potential of Au^+^ reduction to Au is higher in the presence of OA [40, 41]. The primary limiting factor for constant electron transfer rate would be the OA reagent. The relative concentration of the Au microwires obtained was > 50 µg/mL. This method can also be applicable for the synthesis of other metal microwires as long as the chemical combination is suitable and correct.

### Morphological properties of as-synthesised and commercial Au microwires

**Fig. 2:**
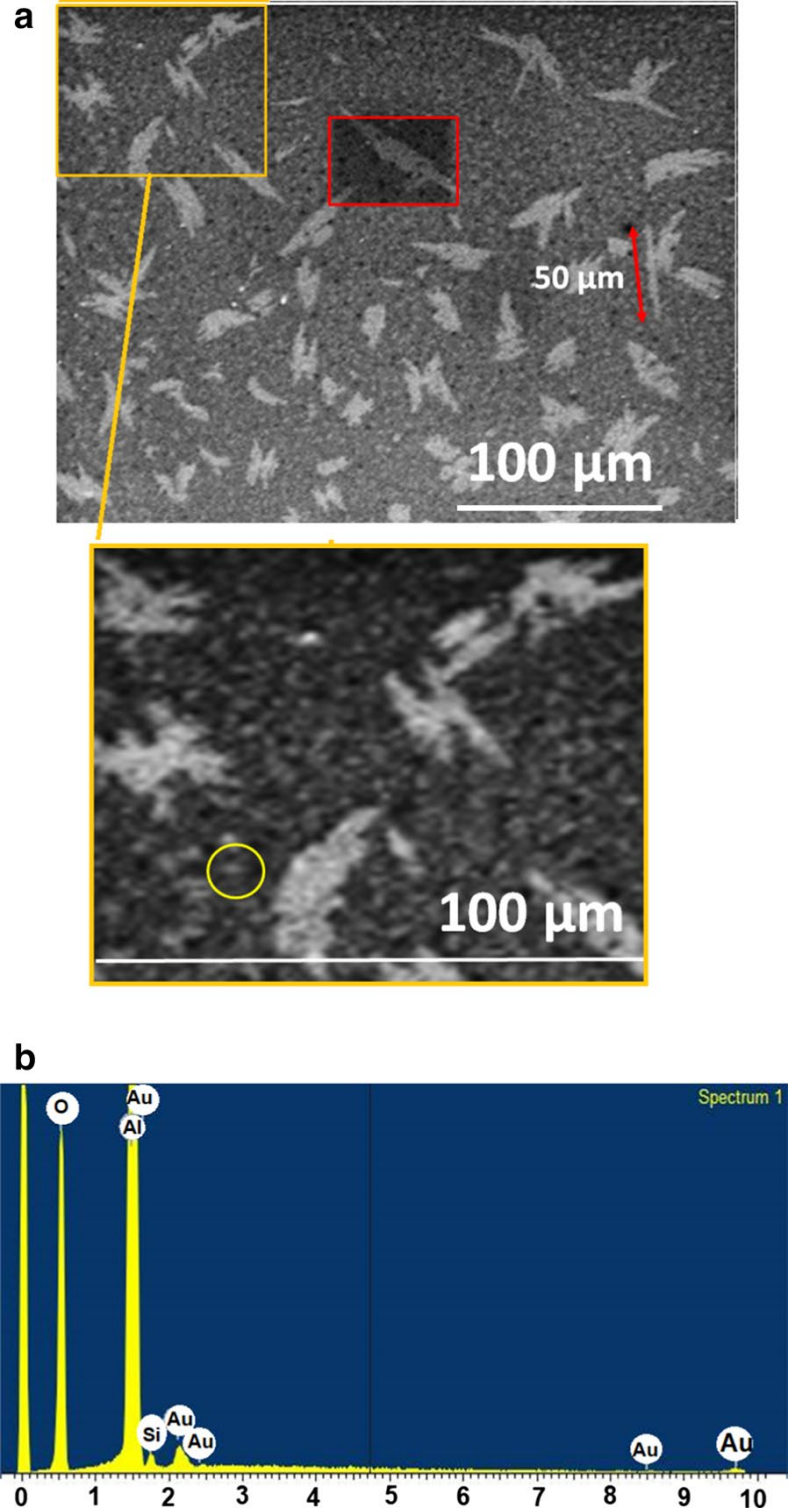
**a** The representative FESEM micrograph of the as-synthesised AuNWs. The enlarged figure (yellow rectangular) reveals the parallel self-assembly arrangement of close-packed AuNWs bundles. The small parallel bundle highlighted in yellow circle showed that the bundle of the microwires has a diameter size of ~ 200 nm. **b** The EDX spectrum analysis of the as-synthesised AuNWs recorded for the selected area of AuNWs (red rectangle) taken from image **a**

The as-synthesised Au microwires samples obtained were characterised for their morphological properties by standard techniques. The morphology, distribution and purity of the as-synthesised Au microwires verified by FESEM are shown in Fig. 2a. For a 24 h reaction, the resultant colloid colour is dark violet which indicates extended filament-like structures. The Au microwires tend to self-assemble into 1-D networks and forming closely packed parallel structures. The use of negatively charged TIPS in the synthetic reaction at room temperature controls the acceleration of the process [42, 43]. The size distribution of parallel Au microwires bundles was difficult to measure. Higher magnification of the assembled ultra-thin Au microwires was unachievable due to their highly sensitive towards the electron beam which resulted in melted microwires within a few seconds of exposure. However, it is expected that the Au microwires possess diameters of approximately larger than 100 nm with an aspect ratio (length-to-width ratio) above 1000 nm. In this case, the Au microwires tended to form a stack of parallel bundles (yellow rectangular) which then self-assembled into 1-D network structures over macroscopic distances through a spontaneous directional aggregation that occurs during solvent evaporation. The directional aggregation is typically formed via oriented attachment in which Au microwires are permitted to fuse as the chemical potential between each chain is different. Therefore, the smoothing extension process to interconnect the of the microwires takes place through diffusion. The Au microwires exhibit higher stability in a polar solvent which is favourable for the subsequent immobilisation of biomolecules.

The primary objective of this work is to produce Au microwires with high aspect ratio and analyse the elements of samples with the quantitative and qualitative analyses properties. Figure 2b shows the element composition of obtained Au microwires samples using EDX. The EDX point measurements were carried out for an accurate estimation of Au amount present in each sample. For as-synthesised Au microwires, the amount of Au is at the level of 98% as the major element in the sample with the remaining amount is Si (substrate). The presence of high purity Au has resulted from homogeneous nucleation of metallic Au as spherical clusters and hence determined the growth of 1-D microwires. This condition proves that in the presence of TIPS, Au seeds have rapid kinetics in the formation of microwires.

**Fig. 3:**
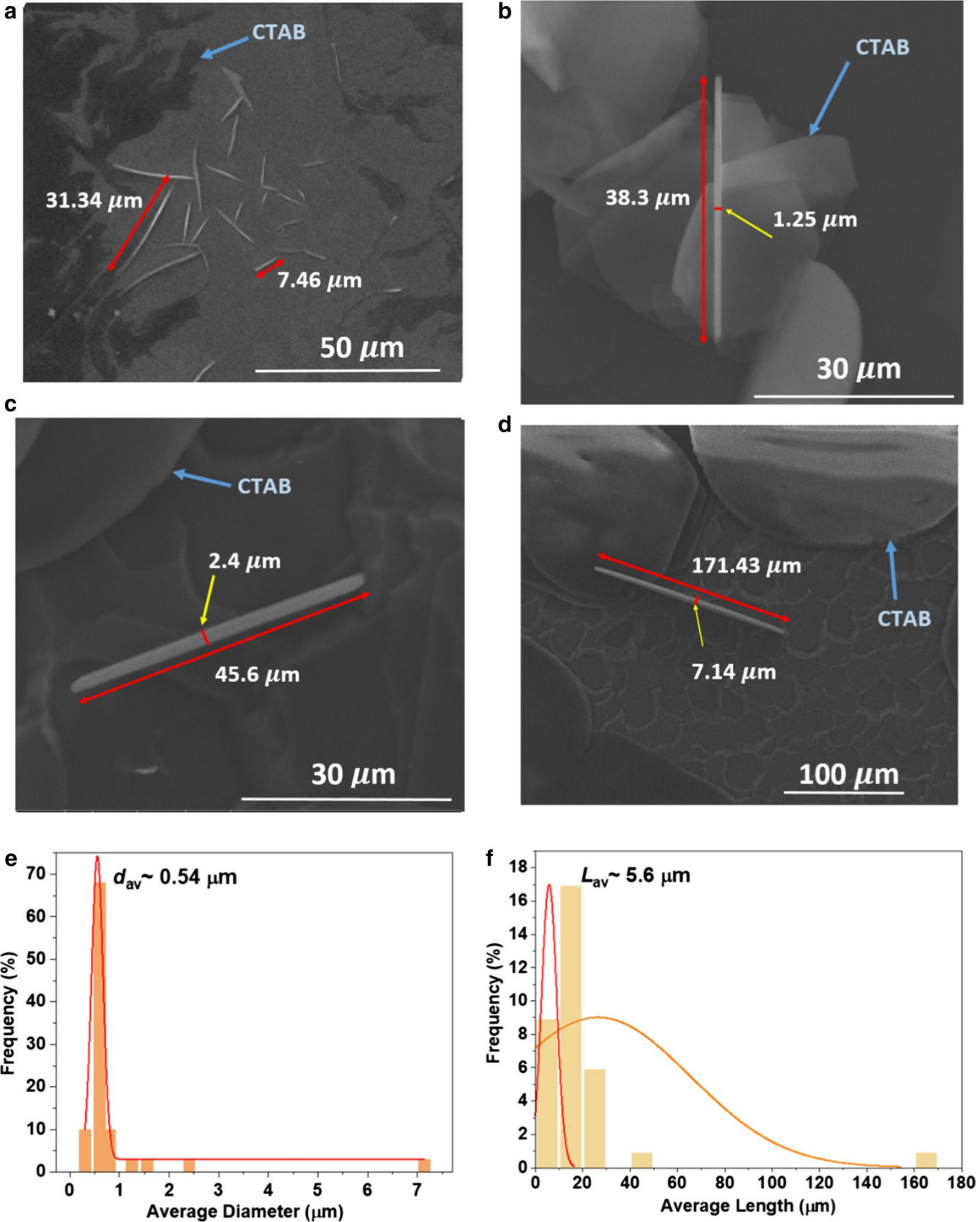
**a** Representative of FESEM images of sonicated commercial Au microwires with CTAB as a capping agent. **b**–**d** Indicate the individual Au microwires. **e** The corresponding diameter and **f** length distributions of the commercial Au microwires in an aqueous solution of CTAB. The average diameter and length of the Au microwires are 0.54 and 5.6 µm, respectively, indicated by the Gaussian plot (red line)

On the other hand, the representative FESEM images of commercial Au microwires suspended in CTAB aqueous solution are shown in Fig. 3. It has been observed that the network formed does not assemble into ordered microstructures (Fig. 3a) as seen in the as-synthesised Au microwires sample beforehand. Instead, the commercial Au microwires are randomly self-gating forming junction connection. Some of the Au microwires showed thicker diameter with reduced lengths as demonstrated in Fig. 3b, c. The Au microwires can be as long as 171 µm (Fig. 3d).

One can see that the diameters of the microwires were averagely thin and approximately $d_{av}$ ~ 540 nm as indicated in Fig. 3e. Interestingly, the $d_{av}$ specified by the supplier is ~ 30 nm. It is common to have different values of diameter and length from the specified information properties provided by the supplier as they are in a discrete condition and slight changes in the physical handling, as well as the time storage, would affect the properties of the sample. Apparently, Au, in its nature, is quite malleable [44–47]. Thus, it facilitates the aggregation, which also correlates to the changes of their stability property. CTAB is used to minimise the aggregation, but it would not be able to halt the entire aggregation of the Au microwires. In this case, CTAB is considered as an effective stabiliser which proved by the average diameter, $d_{av}$ distribution provided in the histogram (Fig. 3e). The $d_{av}$ distribution is dominantly conquered by the 540 nm based on the highest per cent frequency distribution (68%), while the larger $d_{av}$ microwires ($d_{av}$ ~ 1.25 to 7.14 μm) with the per cent frequency is only 3% measured by the Gaussian distribution. This indicates that the 540 nm of $d_{av}$ majorly contributes to the overall properties of the sample.

The average length of microwires, $L_{av}$ typically remained the same caused by the similar diffusion of intense high energy ions in the surrounding. The $L_{av}$ polydispersity distribution of the commercial Au microwires is shown in Fig. 3f. The narrow length distribution range (up to ~ 30 µm) indicates that the commercial Au microwires were stable and monodispersed. The wide distribution range is due to the more considerable length variation, which is up to ~ 171 µm.

It shall be noted that the cloudy region encircling Au microwires is due to the strongly coupled capping action of CTAB upon the evaporation of the solvent, leaving the residue behind. Since cationic CTAB surfactant consists of long carbon atomic chain [48–50], it functions as space-filling of secondary material that fills up the gaps between the Au microwires and maintains the individual distribution of Au microwires. The FESEM micrographs distinctly manifest dissimilarity of CTAB grain with Au microwires, exhibiting large-sized flake grains (marked with the blue arrow in Fig. 3). As stated in few reports [51–53], the tertiary ammonium ion cationic headgroup in the presence of bromide anion as a counterpart in microwire solution results in higher binding affinity and leads to more stable bilayer on the microwire surface. The addition of CTAB micelle helps to stabilise Au microwires when the equilibrium condition is achieved through the occupation some of the surface areas caused by the increased in the aggregation number of CTAB micelle. This stabilisation effect, while making them soluble, can be an inhibitor of subsequent self-assembling process.

### Cytotoxicity evaluation of as-synthesised and commercial Au microwires

The proof-of-concept on the cytotoxicity of the sensors-based microwires requires long acquisition times. Some of the microwires conventional synthesis protocols, such as the reduction of Au precursor in the presence of capping agent can introduce toxic materials from surface conditioning and chemistry [36, 54, 55]. These including surface charge and capping stabilisation without ‘green’ physical approach profited from natural microstructure generation. Additionally, the metal itself can produce toxicity and thus, being unfavourable for *in-vivo* applications. Taking into consideration the unique advantages of Au microwires, different capping interface, these types of Au microwires exhibits a significant effect in microbial viability. For this reason, we examined the potential cytotoxicity propensity for commercial and as-synthesised of Au microwires.

**Fig. 4:**
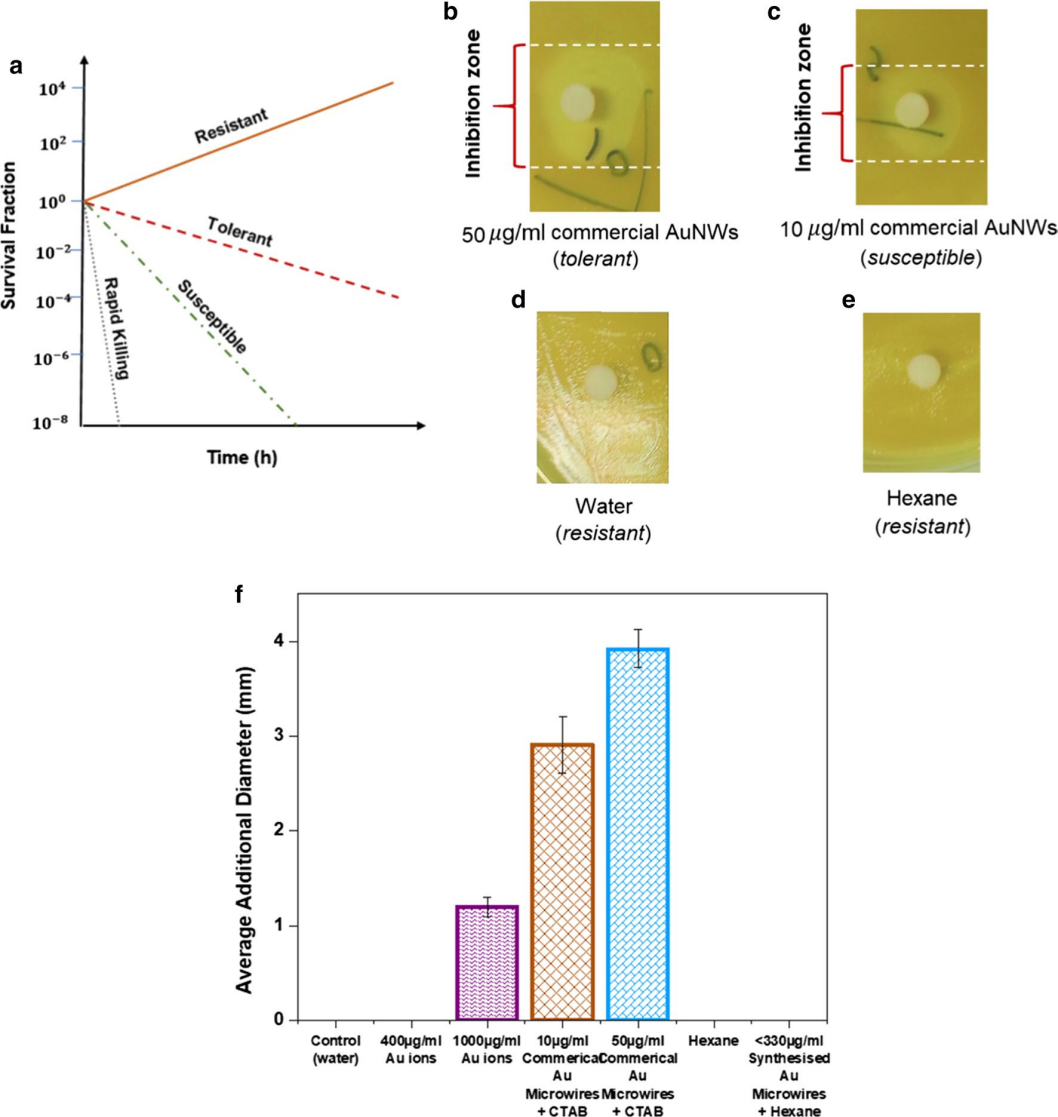
The representative image of *E. coli DH5α* responses to the Au microwires solutions. The disc diffusion assay results with *E. coli DH5α* cells (100 µL) on LB agar overlay. **a**, **b** Indicate the white dotted lines mark the diameter of the white zone where the growth of *E. coli DH5α* is prevented by the Au microwires (inhibition area). **a** Is a tolerant strain with **b** is the susceptible strain. A slightly larger zone of inhibition is seen for tolerant strain due to growth delayed. **c**, **d** Are referring to control sample of water and hexane solutions, respectively. Both show resistant strain. **e** Schematic illustration on the differences between the fraction of surviving bacteria for resistant, tolerant, susceptible and rapid killing strains. **f** Disc diffusion measurements obtained using several concentrations of Au ions and both the as-synthesised and commercial Au microwires. The bars represent one standard error

To further demonstrated the cytotoxicity tolerance in as-synthesised and commercial Au microwires samples, the commercial bacteria *E. coli DH5α*, an engineered non-pathogenic bacterial strain is extensively used as a lab cytotoxicity microbial model system. Two types of cytotoxicity test were carried out. The first is the disc diffusion assay. This method is performed to detect cytotoxicity by inducing a gradient of concentration (Fig. 4a) around a disc (known as inhibitory effect) loaded with Au microwires. The inhibitory effect among the *E. coli DH5α* strains exhibited different outcomes. The ratio of the ring area (measure in mm) is directly proportional to the sample toxicity (white lines) as indicated in Fig. 4b, c. The results show that the gradient of commercial Au microwires suspended in CTAB halts the growth of bacteria and creates an inhibition zone around the disc in both the 10 and 50 µg/mL samples. Lower inhibition zone was found for 1000 µg/mL Au ions. For these types of Au-based suspensions, the largest inhibition zone reached 3.9 ± 0.2 mm, 2.9 ± 0.3 mm and 1.2 ± 0.1 mm for 50 µg/mL commercial AuNWs-CTAB, 10 µg/mL commercial Au microwires-CTAB and 1000 µg/mL Au ions, respectively. Non-toxic samples with no inhibitory effect which refer to water (Fig. 4d) and hexane (Fig. 4e) samples. A similar observation was found for 400 µg/mL Au ions and < 330 µg/mL as-synthesised Au microwires suspended in hexane. The six samples used to observe the average diameter of inhibition zones containing Au-based solutions is presented in Fig. 4f.

The size of as-synthesised Au microwires prepared with growth solution contained Au microwires with a smaller length. Since hexane is a non-polar solvent, it has no charge. Thus, the conjugated surface of Au microwires with negatively charge of the hydrophobic OA, yield a negative surface charge of the Au microwires system [34, 56]. As a result, no electrostatic interactions due to absent of different charges in the solution between the cell surface and as-synthesised Au microwires suspended in hexane. As generally accepted, CTAB alone is highly toxic cationic surfactant [57–59] and the conjugate systems of Au microwires-CTAB are proven to still contribute to toxicity to culture cells especially when the elevated concentrations of CTAB are present (50 µg/mL commercial Au microwires-CTAB). With increasing the amount of Au microwires-CTAB solution, the cytotoxicity of the sample is increased caused by the nonspecific binding tendency of CTAB to negatively charged cell surfaces by electrostatic interactions [57, 60]. Once the interaction with the cell occurs, it forms blebs and holes on the cell and leading to cell death evident by the increase in the diameter of the ring zone. Moreover, the data also suggest that the abilities to induced cell death are also significant regardless of the aspect ratio of the Au microwires, in this case, the aspect ratio is constant for both 10 and 50 µg/mL commercial Au microwires-CTAB solution. To minimise the cytotoxicity effects in Au microwires-CTAB, the interaction between the membrane cell and CTAB should be blocked by using protein types of coating on the bilayer surface of CTAB. Meanwhile, a similar trend of nonspecific binding also observed when Au ions are incubated with the cell. The nonspecific binding was evident by the increased of the ring zone when exposed to the concentration of the 1000 µg/mL Au ions. This nonspecific binding did not take place with 400 µg/mL Au ions might be due to the reduced content of positively charge Au ions, which in turn, decreases electrostatic interaction between the Au ions and negatively charged cell membrane. These results affirm cytotoxicity potency of commercial Au microwires-CTAB and Au ions compared to as-synthesised Au microwires suspended in hexane.

**Fig. 5:**
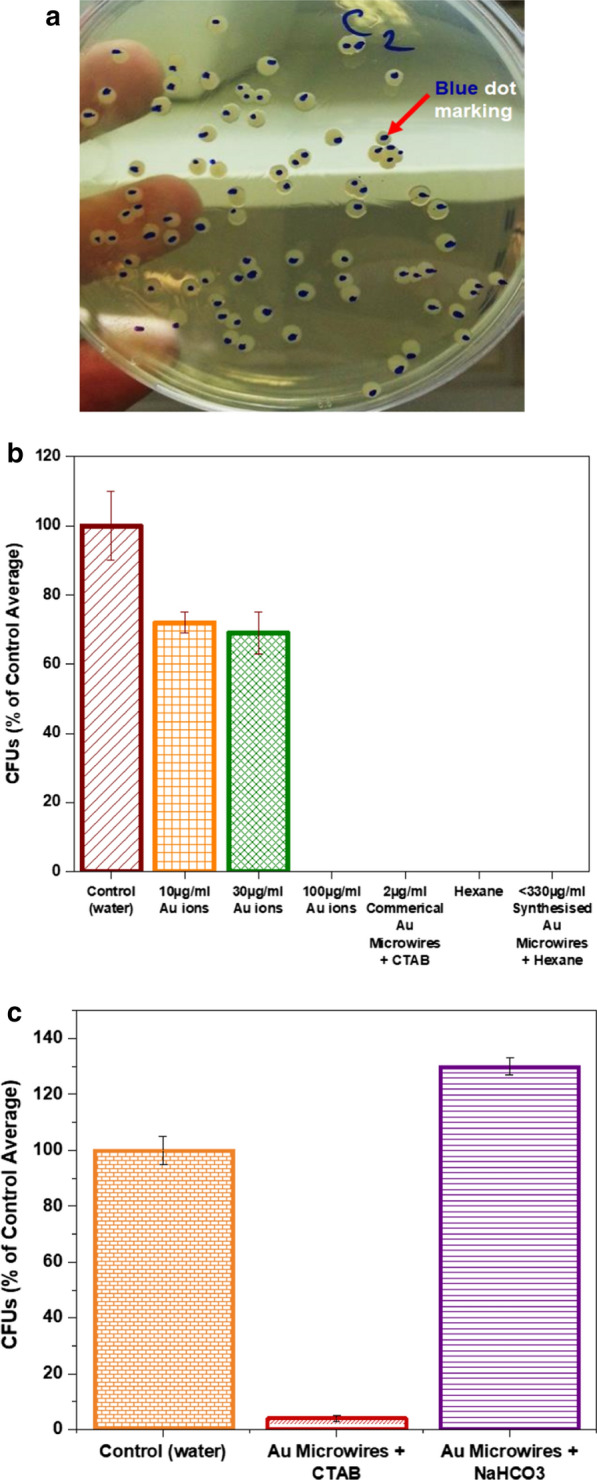
**a** The representative of Au microwires antimicrobial CFU enumeration based on the individual colonies of *E. coli DH5α* formed after 24 h. Blue marker was used to mark the colonies with dots marking at the bottom side of the petri dish. **b** The number of bacterial colonies (%) appeared in agar plated treated with control (water), 10, 30 µg/mL and 100 µg/mL of Au ions, 2 µg/mL of commercial Au microwires suspended in CTAB, hexane and < 330 µg/mL as-synthesised Au microwires in hexane. **c** Activity of antimicrobial CFU assay of Au microwires capped with CTAB and NaHCO_3_ after 24 h of plate-incubation. Water is used as a control sample

For further validation of cytotoxicity detection on commercial and as-synthesised Au microwires, the CFU enumeration is carried out after 24 h incubation (Fig. 5a). As evident from Fig. 5b, the CFU enumeration for 10 µg/mL Au ion measured viability of 72%. At 30 µg/mL Au ion approximates value were almost similar to the other one giving rise to 69%. Cell-viability assessed by CFU remained at 100% on the control (water) sample. Compared with 10 and 30 µg/mL of Au ions, the cell incubated with a higher concentration of Au ions (100 µg/mL) was observed to be 0% cell viability. In the case of 2 µg/mL of commercial Au microwires suspended in CTAB, < 300 µg/mL as-synthesised Au microwires and hexane also, 0% were viable for all in CFU enumeration. Pure hexane, water and the ionic Au at three different concentrations (10, 30 and 100 µg/mL) were used as negative controls. Assessment at 96 h was likely to be optimal for Au microwires strain. Indeed, the effect of both commercial and as-synthesised Au microwires along with a high dosage of 100 µg/mL Au-based ion seemed strong antibacterial agent in this assay. Considering the high amount concentration use for as-synthesised Au microwires, it is suggested that the Au microwires work markedly better, meaning presents less bacteria toxicity than Au ions (tenfold lower in concentration) as happened to other metallic materials.

Endowing the high antibacterial potency or toxicity effect based on the viability results for both types of Au microwires at those particular concentrations, another study is carried out to compare the inhibiting capability of commercial Au microwires-CTAB with Au microwires-sodium bicarbonate (NaHCO_3_) towards the cell growth. The focused study is purposely done on the commercial Au microwires-CTAB since a relatively low amount of sample concentration used proved to affect the cell viability significantly. To further clarify the inhibitory antibacterial activity induced by the CTAB layer, commercial Au microwires were purified from CTAB capping layer and re-dispersed in 2 mM NaHCO_3_. Cells grown in LB media with Au microwires-CTAB and Au microwires-NaHCO_3_ with an approximate volume of 250 µL for both were observed. Water served as the standard control. The cell-microwire-LB solutions were approximately 5 µg/mL, incubated for 90 min and was serially diluted to 105 times of the initial concentration with the set to dispense for 100 µL onto plates. As shown in Fig. 5c, CFU enumeration showed 4% and 130% of capture efficiency for Au microwires-CTAB and Au microwires-NaHCO_3_ solutions, respectively. The low CFU in the CTAB solution was found to be threefold lower than in NaHCO_3_, indicating that the toxicological effect of CTAB is relatively high, which significantly displayed susceptible inhibit growth of *E. coli* cells. NaHCO_3_ had a low inhibitory effect.

The results demonstrated that surface modification is the decisive factor governing the cytotoxicity of Au microwires. CTAB proved the capability to induce *E. coli DH5α* cell death and destroy bacteria growth. As regards to the surface modification, the Au microwires capped with NaHCO_3_ (well-known as baking soda), indicated minimal toxicity effect and had almost no effect on cell viability in an alkaline environment. The alkaline environment retarded the activity of the toxicity which inhibits the cellular uptake. When combining with NaHCO_3,_ the acidic environment that usually leads to cell death is halts and decreasing the risk of adverse effects and toxicity.

It is believed that the primary toxicity mechanism of the Au microwires-CTAB was via the activation of intracellular cell damage, which rigidly controls the per cent of cell survival. As the cell tries protecting itself from toxicity created by the molecule, other existing plasma proteins will adsorb on the surface of microwires. The microwires eventually penetrate the cells after adjusting the interfacial properties of the adsorbed protein shell. However, some of the adjustments are not always a success as not all modified Au microwires surface would able to enter the cells effectively. The proper conjugated Au microwires able to recognise the proteins on the cell membrane. Thus, adsorption of the protein to the surface of the microwires mediate the direct penetration of microwires. Typically, smaller Au microwires are frequently deemed as more toxic due to the ability to fully penetrate at intracellular locations (e.g., nucleus) which is not able to get through by larger microstructures. Hence, since cytotoxicity was controlled by the physicochemical surface properties (surface charge number and dimensions) of Au microwires, it is crucial to employ riskless surface modification reagent to govern the biological effects of microwires to ensure the safety of microstructures used in medical applications.

## Conclusions

As discussed, the synthesis of Au microwires has tailored microwires that formed the irregular chain-like oriented assembly. These Au microwires in hexane solution are almost similar in average length and ten times smaller in diameter size as compared to commercial Au microwires suspended in CTAB solution. However, they are unstable at relatively high temperatures, melting within a few seconds upon the heat exposure. The anionic polymer of OA was used as a capping layer to protect as-synthesised Au microwires from aggregation, which also render them to be biocompatible. The feasibility of the Au microwires reveals that the functionalised Au microwires in the present of OA was able to avoid cytotoxicity to the membrane cell. On the other hand, the commercial Au microwires in high crystallinity state are stable at elevated temperatures and the toxicity responds observed is mainly caused by the CTAB capping layer and not due to the aspect ratio of the Au microwires. In fact, the pristine Au microwires alone was generally non-toxic and proved to have low cytotoxicity when capped with NaHCO_3_ upon eliminating the CTAB layer on the microwires. These different surface modifications have shown that the cytotoxicity of Au microwires is closely related to the original physicochemical properties of the capping layer based on the in vitro cell investigation results. Hence, the as-synthesised Au microwires and the Au microwires-NaHCO_3_ could be considered as the optimal options for integrating and implanting in MC biosensor devices, including high-throughput applications.

## Supplementary information

**Additional file 1.** The assembling microwires on the top surface of the microscopy probe is also evaluated but not discuss further in the present study and only included in the Supplementary Materials section.

## Data Availability

Data are available on request due to privacy or other restrictions.
