# Supplementary material for: Synthesis, characterisation and cytotoxicity of gold microwires for ultra-sensitive biosensor development
Source: Microb Cell Fact. 2021 Feb 17;20:46. doi: 10.1186/s12934-020-01478-y (PMC7888188; doi:10.1186/s12934-020-01478-y)
Supplement: Supplementary file 1 — Additional file 1. The assembling microwires on the top surface of the microscopy probe is also evaluated but not discuss further in the present study and only included in the Supplementary Materials section. [file 12934_2020_1478_MOESM1_ESM.docx]

**Synthesis, Characterisation and Cytotoxicity of Gold Microwires for Ultra-Sensitive Biosensor Development**

^1^Nurul Akmal Che Lah, ^2^Robert Gray & ^3^*Sonia Trigueros

^1^*Innovative Manufacturing, Mechatronics and Sports Lab (iMAMS), Faculty of Manufacturing and Mechatronics Engineering Technology, University Malaysia Pahang, 26600, Pekan, Pahang, Malaysia.*

^2^*University College London, Gower St, Bloomsbury, London, WC1E 6BT, United Kingdom.*

^3.^*Department of Zoology, University of Oxford, OX1 3PS, Oxford, United Kingdom.*

*****Correspondence: *Sonia Trigueros*

email: [*sonia.trigueros@zoo.ox.ac.uk*](mailto:sonia.trigueros@zoo.ox.ac.uk)

**Assembly on Cantilever**

Au microwires were assembled onto a cantilever surface by Au microwires -ethanol droplets evaporation method. Aluminium-coated cantilevers (Concentric GmbH-Switzerland) with 400 $\mu$m long, 1 $\mu$m thick and 100 $\mu$m wide were used in the process. 20 µl of the colloidal solution was sonicated using an ultrasonic bath for 15 min. A 5 μl drop of the solution was added onto the cantilever surface. After letting the dropping air dry for 24 h, Au microwires adhered to the surface. The Au microwires assembly was imaged using FESEM.

Integrating Au microwires onto a cantilever is crucial for the exploration of a multifunctional MC biosensor. To functionalise the cantilever, Au microwires were attached to an aluminum coated AFM cantilever surface. The surface of the cantilever was covered with Au microwires solution, and the microwires on the surface repel each other and assemble. In this case, for the functionalisation process, we used commercial Au microwires and the evaporation process described in material and methods. The resulting attachment between the surface of the cantilever and Au microwires is shown in **Figure S1**. This assembly is adequate for MC biosensor functionalisation viability. Here, we demonstrated a direct depositing of Au microwires and its binding tendency onto the cantilever.

******

**Figure S1**: FESEM micrograph of commercial Au microwires deposited on the aluminium-coated AFM cantilever surface. The formation of the scaffold is due to the coffee-ring effect during the evaporation process. Au microwires are deposited along the solid-liquid contact line.

The undoubted benefit of the MC surface Au micro-structuration is the expansion of the cantilever limited sensitive range. It is well-known that one of the utmost drawbacks of micro sensors is their low dynamic range. A higher-performance functional surface of Au microwires could increase the binding capacity, and therefore, increasing the sensor detection to a broader range of molecule type and concentrations.

Minimising the thickness and width of the sensing element is also essential to retain an excellent signal efficiency from a strain-sensing component of MC sensor. The thickness of the Au microwires is in the range of 80 – 100 nm and width of 2.47 mm, which rules out the employment of sensors made of conventional self-sensing elements including Si and PZT. The small diameter with a much longer length of Au microwires permits the fabrication of strain sensors on small MCs without significantly affecting their dynamic properties. Also, the achievable wider sensor length is crucial in maintaining low resistance and a very high sensitive detection bandwidth. Altogether, the fabrication example in this study highlights the potential multifunctional design of Au microwires -based MC probes. Also, take into account that bulk fabrication of the MC probe at the larger scale could be possible.

Although, the Au microwires the better to increase the microsensor sensitivity, commercial Au microwires are ideal for the MC deposition. Therefore, more studies need to be undertaken to elucidate the synthesis of thinner and stable Au microwires with both high defect characteristic and intrinsic quality to allow better operation and function at a higher temperature.
